# Nasal trigeminal projections and medullary dorsal horn neuronal activation during voluntary diving in rats

**DOI:** 10.3389/fphys.2025.1669864

**Published:** 2025-10-29

**Authors:** P. F. McCulloch, M. Margosiak, H. Namburi, K. Kernosek, K. M. DiNovo

**Affiliations:** ^1^ Department of Physiology, College of Graduate Studies, Midwestern University, Downers Grove, IL, United States; ^2^ Chicago College of Osteopathic Medicine, Midwestern University, Downers Grove, IL, United States; ^3^ Biomedical Sciences Program, College of Graduate Studies, Midwestern University, Downers Grove, IL, United States; ^4^ College of Pharmacy, Midwestern University, Downers Grove, IL, United States

**Keywords:** wheatgerm agglutinin, trigeminal nerve, MDH, trigeminal nucleus, diving, Fos

## Abstract

The diving response, an autonomic reflex characterized by apnea, bradycardia and increased peripheral vascular resistance, is initiated when animals submerge underwater. Neurons located within the trigeminal nucleus caudalis, specifically the ventral medullary dorsal horn (MDH), become activated and produce Fos after repetitive diving. It is assumed nerves innervating the nasal passages are important for activating these neurons during diving. The present study investigated the anatomical route by which nasal stimulation during diving can produce activation of MDH neurons. In rats trained to repetitively dive underwater, transganglionic tracer WGA was injected into the nasal passages or nerves innervating the nasal passages. Immunohistochemistry revealed the ventral superficial laminae of the MDH, between the pyramidal decussation and obex, receives central terminations from the nasal passages, superimposing the location containing neurons activated by repetitive diving. After WGA injection into the nasal passages, colocalization of WGA and Fos-positive neurons increased significantly from 4.6 ± 2.1 in non-diving rats to 32.3 ± 10.6 in diving rats. After WGA injection into the anterior ethmoidal nerve (AEN), colocalization of WGA and Fos-positive neurons increased significantly from 10.3 ± 3.2 in non-diving rats to 29.0 ± 5.2 in diving rats. Additionally, diving rats exhale air bubbles from their nose during diving and allow water to enter their nasal passages while underwater. We conclude sensory information projecting from the nasal passages via the AEN likely activates MDH neurons and induces them to produce Fos during repetitive diving. We are less confident about the role of the nasopalatine nerve.

## 1 Introduction

The diving response, an autonomic reflex characterized by apnea, bradycardia and increased peripheral vascular resistance (Butler and Jones, 1997; Panneton, 2013; Fitz-Clarke, 2018), is initiated when animals submerge underwater. Information involved in initiating this cardiorespiratory response likely is processed by neurons located within the spinal trigeminal nucleus caudalis (also known as the Medullary Dorsal Horn (MDH) due to its morphological similarity with the spinal dorsal horn (Gobel et al., 1988; Capra and Dessem, 1992)). When rats voluntarily dive underwater, neurons located within the ventral MDH are activated, as shown by their expression of Fos (McCulloch, 2005; Panneton et al., 2010a; Panneton et al., 2012; McCulloch et al., 2016). The activation of these MDH neurons is thought to constitute second-order afferent processing that is part of the brainstem circuit producing the diving response (Panneton et al., 2006; Panneton, 2013; McCulloch et al., 2018).

McCulloch et al. (2018), using the transganglionic tracer wheat germ agglutinin (WGA), found that afferent fibers from the nose and nasal passages primarily project to the ventral MDH. Additionally, the anterior ethmoidal nerve (AEN), a branch of the ophthalmic division of the Trigeminal Nerve (Cranial Nerve V; (Shankland, 2001; Prendergast, 2013)) that innervates the internal nasal passages, sends its central projections to the ventral portion of the MDH in the rat (Anton and Peppel, 1991; Panneton et al., 2006; McCulloch et al., 2018), cat (Lucier and Egizii, 1986), and muskrat (Panneton, 1991). The close anatomical similarity between the location of activated MDH neurons during diving and central terminations of nasal afferents has been noted previously (McCulloch, 2005; Panneton et al., 2010a; McCulloch, 2012; Panneton, 2013; McCulloch et al., 2016; McCulloch et al., 2018), which has led to the assumption that nerves innervating the nasal passages are important for activating secondary neurons within the MDH during diving. However, this intriguing anatomical similarity has never been specifically investigated until now. Examination and verification of this assumption provided the scientific rationale for conducting this study.

In the present study we investigated the anatomical route by which stimulation of the nasal passages during diving can produce activation of MDH neurons. In rats trained to repetitively dive underwater, we injected WGA into the nasal passages or nerves known to innervate the internal nasal passages. After having the rats repetitively dive underwater to induce MDH neurons to produce Fos, we then used immunohistochemistry to identify activated MDH neurons that receive afferent projections from the nasal passages. We found that sensory information projecting from the nasal passages via the AEN is likely responsible for activating MDH neurons and inducing them to produce Fos during repetitive diving. We also investigated the behavior of rats diving underwater, and found that rats exhale air bubbles from their nose during diving, and, counterintuitively, allow water to enter their nasal passages while underwater.

## 2 Methods

All studies were approved by the Midwestern University IACUC. Male Sprague-Dawley rats (N = 40) were purchased from a commercial vendor (Envigo, Indianapolis IN) at 3 weeks of age (approximately 25 g each) and were housed within the Midwestern University Animal Facility. Animals were kept in pairs, except after WGA tracer injections or surgery (see below) when they were caged singly, in accordance with the NIH guidelines for the care and use of laboratory animals. Rats were provided with food and water *ad libitum* and were provided with cage enrichment. Rats progressed through swim and dive training (see below) until they were adults (300–350 g).

### 2.1 Swim and dive training

**FIGURE 1:**
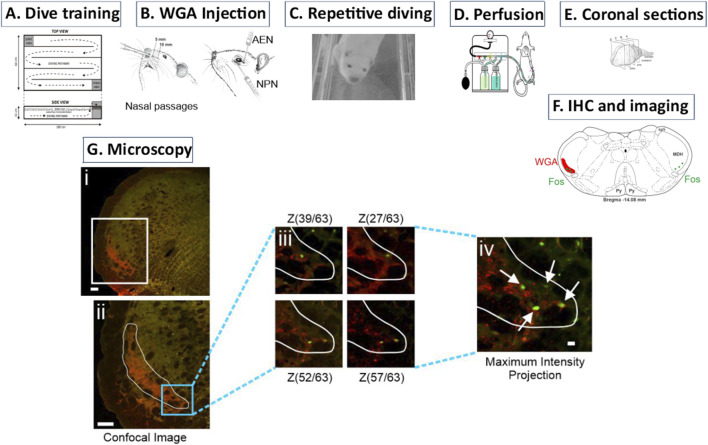
Description of methodology. **(A)** Over 10 weeks rats were trained to first swim and then dive through an underwater maze. **(B)** After dive training was completed, WGA was injected into (left) the nasal passages or (right) individual trigeminal nerves (anterior ethmoidal nerve (AEN) or nasopalatine nerve (NPN)). **(C)** On the day of the experiment, rats repetitively dived voluntarily through the underwater maze to activate neurons within the medulla that are part of the diving response. These activated neurons produced Fos protein. **(D)** After completion of diving, rats were sacrificed and perfused with paraformaldehyde. Fixed brains were removed and stored. **(E)** The brainstem and rostral spinal cord was then cut into 50 µm coronal sections. **(F)** Using immunohistochemistry (IHC), brainstem sections were tagged for both WGA and Fos. Within the medullary dorsal horn (MDH), WGA labelling extended from the pyramidal decussation caudally to the obex rostrally. **(Gi)** Confocal images were taken of each left brainstem hemisection to create a Z-stack. **(Gii)** A region of interest (ROI) was drawn to outline the extent of the WGA labeling. **(Giii)** Within this ROI, the Z-stack images were individually inspected to identify Fos-positive neurons at differing focal planes. Note that different Fos-positive neurons appear at different Z-stack layers within the 50 µm coronal section. **(Giv)** A maximum intensity projection image of all Z-stack focal planes was then used to count the total number of individually identified Fos-positive neurons within the MDH WGA ROI.

Cohorts of rats (10–12 rats per cohort) were trained to first swim and then to dive under water through a 5 m long water maze constructed from Plexiglass (Figure 1A). For details of the training procedure see McCulloch (2014). Briefly, the rats were trained 5–6 times per week for 10 weeks, with 3–5 trials per day and with 5 min separating each trial. The water in the maze was changed for each training session, and was maintained at 30 °C ± 2 °C. The rats were dried with a towel between each trial. Initially the rats were trained to swim to the finishing area of the Plexiglas maze. The length of the swimming distance was increased during each training session until the rats could swim the entire 5 m of the maze. Once this was completed, a vertical barrier that blocked the surface of the swimming channel was inserted into the maze, causing the rats to dip under the barrier to engage in their first dive. Gradually horizontal subsurface barriers were inserted into the maze channels which extended the distance the rats had to dive underwater before resurfacing and swimming the remaining distance to the finishing area. Once the dive training was completed, the rats could dive underwater through the entire 5 m maze in 10–15 s.

### 2.2 Role of AEN during repetitive underwater diving

To determine the role of the AEN during submergence, and after mastering the repetitive diving protocol, some rats received bilateral sectioning of the AEN, while other rats received sham AEN surgery. After a dorsal skin incision, the AEN was accessed within the orbit by laterally retracting orbital contents. In all animals receiving AEN sectioning, a 1 mm piece of AEN was excised and produced for inspection to confirm the denervation. For sham surgeries the AEN was isolated but left intact. All rats were given the analgesic Ketoprofen (5 mg∙kg^−1^, s.c.) immediately following surgery and again 24 h later. Surgical recovery lasted 5 days before rats returned to diving. One cohort of diving rats (N = 10) was closely observed to determine whether rats expel air bubbles from their noses while underwater, both before surgery and after sham (N = 4) or real (N = 6) AEN surgery.

### 2.3 Injection of WGA into nasal passages and trigeminal nerves

The transganglionic tracer WGA was used in two ways to determine the extent of the central projections of the afferent innervation emanating from the nose and nasal passages (N = 30; Table 1). Following the procedures described by McCulloch et al. (2018) WGA was either injected into the nasal passages, or directly injected into nerves known to innervate the nose and nasal passages (anterior ethmoidal nerve [AEN] and nasopalatine nerve [NPN]). Before WGA injection, all rats were anesthetized with ketamine/xylazine (80 mg∙kg^−1^/10 mg∙kg^−1^ i.p.), with supplemental dosages given as needed.

**TABLE 1:** Hemisections containing Fos-positive neurons.

Condition	N	Hemisections containing Fos-positive neurons	Number of Fos-positive neurons within WGA ROI per hemisection
WGA into nasal passages			
No dive control	4	7.5 ± 0.5	4.6 ± 2.1
Diving rats with intact AENs	3	11.7 ± 2.2	32.3 ± 10.6*
Diving rats with AENs cut	3	4.0 ± 0.6	8.4 ± 4.2
WGA into AEN			
No dive control	6	10.2 ± 1.6	10.3 ± 3.2
Diving rats	7	11.0 ± 0.6	29.0 ± 5.2*
WGA into NPN			
No dive control	3	9.0 ± 1.0	23.6 ± 16.3
Diving rats	4	7.8 ± 0.6	26.1 ± 8.1

Number of animals per diving condition (N); average number of left hemisections containing Fos-positive neurons within the WGA, region of interest throughout the rostral-caudal extent of the MDH, per rat after injection of Wheat Germ Agglutinin (WGA) into the left nasal passages, anterior ethmoidal nerve (AEN), or nasopalatine nerve (NPN); and average number of Fos-positive neurons within WGA ROI, per hemisection. * = significantly different from No dive control.

In N = 10 rats, WGA (50% WGA (Vector L-1020) and 50% methylcellulose gel (Sigma M7140)) was introduced into the nasal passages (Figure 1B left). WGA (60 µL total) was injected into the left nasal passages (30 µL each at depths of 5 and 10 mm). In some rats (N = 3) the AENs were sectioned bilaterally immediately before WGA injection by removing a 1 mm piece of each nerve. These animals were given the analgesic Ketoprofen (5 mg∙kg^−1^, s.c.) immediately following surgery and again 24 h later.

In other rats, after a dorsal skin incision, the AEN or NPN was accessed within the left orbit by laterally retracting orbital contents (Figure 1B right). In N = 13 rats 100% WGA (1 µL) was injected directly into the left AEN as it traversed the orbit. In N = 7 rats 100% WGA was injected directly into the left NPN, as it exited the orbit through the sphenopalatine foramen deep to the main bundle of the infraorbital nerve within the orbit (Figure 1B right). WGA injection into the NPN (2.5 µL) was followed by placing a small WGA-soaked cotton ball directly on the NPN for 15 min. Additional cotton balls were carefully placed to prevent possible spread of WGA to other nerves within the orbit. This WGA application procedure was performed twice for each rat.

### 2.4 Diving protocol

Following 4–5 days of WGA transport, experimental rats then engaged in repetitive diving (Figure 1C). In this protocol each rat dived though the maze every 5 min for 2 h, for a total of 24 dives (McCulloch, 2005; McCulloch et al., 2016). Then the rats were placed back in their cage to rest for 1 h to allow neuronal production of Fos. Non-diving control rats did not engage in any diving activities even though they had successfully completed all swim and dive training. All rats were deeply anesthetized with a concentrated pentobarbital solution (Euthasol, Virbac) and then transcardially perfused with saline and then 4% paraformaldehyde (Figure 1D). The brain and rostral spinal cord were removed and placed overnight in a 20% sucrose-paraformaldehyde post-fix solution. Next the brainstem was blocked and then cut from the rostral spinal cord to the inferior colliculus using a freezing microtome that produced 50 μm coronal slices (Figure 1E). Brainstem tissue slices were collected in a 24-well plate and stored in a cryopreservative solution at −20 °C for later immunohistochemistry.

### 2.5 Immunohistological processing

A 1 in 3 series of the stored brain tissue was then used for immunohistochemistry (IHC) to locate the medullary termination of WGA that had been previously injected into the nasal passages or specific trigeminal nerves, and to identify neurons that expressed Fos and thus were activated during repetitive diving. PBS (or PBS with 2% Triton) was used for all immunohistochemical washes between incubations. Tissue was first blocked in 5% normal donkey serum for 1 h. The tissue was then incubated overnight with a rat anti-Fos primary antibody (SYSY 226017). The next day the tissue was blocked for 1 h in 5% bovine serum albumin (BSA). The tissue was then incubated for 2 h with a fluorescent-tagged donkey anti-rat secondary antibody (Invitrogen A21208; green, AF 488). The tissue was again blocked for 1 h in 5% BSA. The tissue was then incubated overnight with a goat anti-WGA primary antibody (Vector AS-2024). On day 3 the tissue was blocked for 1 h in 5% BSA and was then incubated for 2 h with a biotinylated rabbit anti-goat secondary antibody. The tissue was then incubated for 2 h with fluorescent Streptavidin Dylight (Vector SA-5594; red, AF 594). The tissue free-floating in PBS was rearranged into serial order, mounted on slides, and then coverslipped using hard set mounting media (Vector H-1700). This IHC procedure produced red labelling of WGA that identified central projections from the nasal passages or specific trigeminal nerves (AEN or NPN), and green labelling of Fos neurons that identified neurons activated by repetitive diving (Figure 1F).

### 2.6 Microscopy and analysis

Left tissue hemisections were first imaged using a Nikon AX-R confocal attached to a Nikon Ti2 microscope to create Z-stack photomicrographs of WGA labelling (Nikon Elements). Each 50 µm thick section usually yielded 50–70 individual Z-stack images. A maximum intensity projection of the Z-stack images was then created (Figure 1Gi), and from this the extent of WGA terminal projections determined a region of interest (ROI; Figure 1Gii). The individual Z-stack images then were used to determine and confirm the presence of Fos-positive neurons within the WGA region of interest, as Fos-positive neurons were located at different focal planes within the Z-stack (Figure 1Giii). A maximum intensity projection image of all Z-stack focal planes was then used to count the total number of individually identified Fos-positive neurons within the WGA ROI (Figure 1Giv). Note that WGA is visualized in the central terminals of primary afferent nerves, whereas Fos immunoreactivity is located in cell nuclei of neurons, and thus double-labelled neurons (WGA+Fos) were not encountered. Instead, the number of Fos-positive neurons within the WGA ROI was counted. An average of 9 ± 0.57 rostral to caudal left MDH hemisections per animal were used to count the number of Fos-positive neurons within the WGA ROI (Table 1). Although the central projections of WGA injected into the nasal passages were found in other areas of the medulla (i.e., NTS; see Figure 4), Fos-positive neurons outside the MDH were not counted.

Photomicrographs were adjusted with Nikon Elements, and figures were composed and labeled using CorelDraw (Corel). Neuronal data are presented as the number of Fos-positive neurons ± SE within the WGA ROI per hemisection. Statistical differences were tested with Student’s t-tests or one-way ANOVAs (SigmaStat 15.0, SPSS) with p < 0.05 set as the level of significance.

## 3 Results

### 3.1 Repetitive underwater diving

After recovering from the surgery to inject WGA and/or bilateral sectioning of the AENs, rats easily returned to underwater diving. Voluntary dives were usually initiated within 30 s after being placed in the starting chamber, and mean underwater dive duration through the maze was 13.9 ± 0.4 s. Rats sometimes defecated while diving, producing 4.0 ± 1 defecation pellets per rat per 24 dive trials. There were no noted differences in diving behavior between rats with or without intact AENs.

On occasion some rats had slightly bloody noses after completing their dives and while grooming themselves on the finishing area platform. The bloody noses occurred more often during dive training in juveniles, and less often in fully trained adults. There were no differences noted in the frequency or severity of the bloody noses between rats with or without intact AENs. Also, between diving trials and while on the finishing area platform, some rats would submerge their heads under water, presumably exploring their environment. Sniffing movements, both of the air and water, were observed.

**FIGURE 2:**
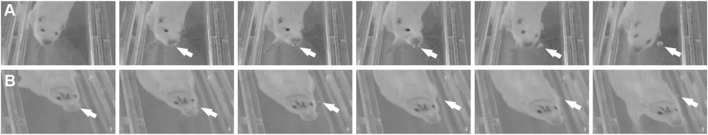
Diving bubbles. As rats dived through the underwater maze, they often expelled air bubbles from their nostrils (arrows). Bubbles were observed both **(A)** with AENs intact and **(B)** after bilateral sectioning of the AENs.

In the cohort of rats used to confirm whether rats expel air out their nose while diving underwater, all 10 rats produced air bubbles while diving, but not all rats produced air bubbles during every dive. Before surgery to bilaterally section the AENs, air bubbles were observed emanating from the nose during 79% of all dives (Figure 2A). For individual rats, the frequency of dives in which bubbles were produced ranged from 57% to 100%. After sham AEN surgery (N = 4), bubbles were observed during 70% of all dives (frequency for individual rats ranged from 33% to 100%). After bilateral AEN surgery (N = 6), bubbles were observed during 83% of all dives (frequency for individual rats ranged from 67% to 92%; Figure 2B). Rats did not produce air bubbles immediately upon submersion. Instead, rats generally expelled air from their noses in the middle half of the underwater pathway, most often when rounding corners between channels but also while diving through channel straightaways.

### 3.2 Central termination of WGA within the medulla

**FIGURE 3:**
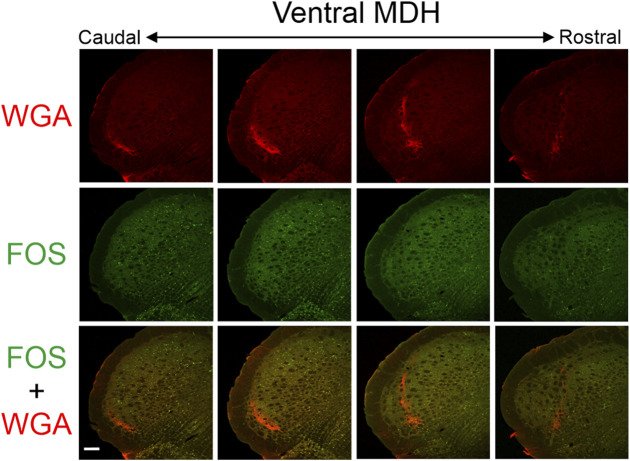
Low magnification confocal images of the left MDH from repetitively diving rats that had WGA injected into their left nasal passages. Images are maximum intensity projections and are arranged from caudal (left) to rostral (right). Top row shows only WGA label. The bright red shows the central termination of nerves that innervate the nasal passages. Note how the label changes orientation when moving from caudal to rostral. Middle row shows only Fos label. Although difficult to see at this level of magnification, Fos-positive neurons have bright green within their nuclei. Bottom row shows an overlay of both WGA and Fos label. Scale bar in bottom left panel = 200 μm for all panels. Images correspond to Figure 1Gi.

**FIGURE 4:**
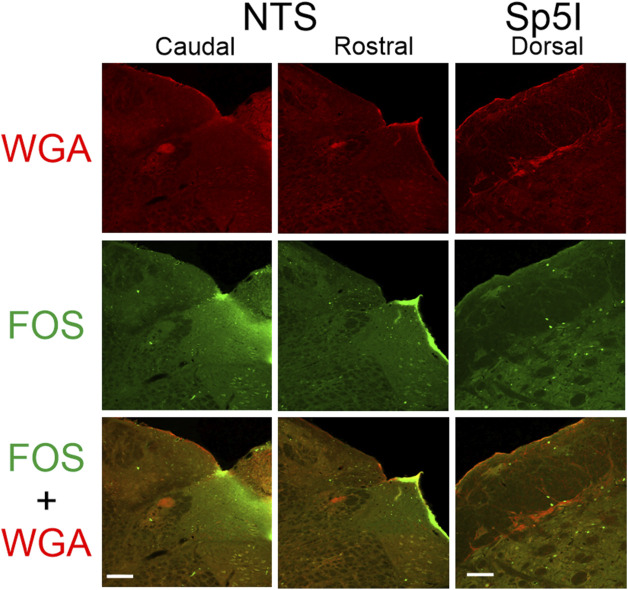
Low magnification confocal images of the left NTS (caudal - left; rostral - middle) and left dorsolateral edge of Sp5I along the spinal trigeminal tract (right) from repetitively diving rats that had WGA injected into their left nasal passages. Images are maximum intensity projections. Top row shows only WGA label. The bright red shows the central termination of nerves that innervate the nasal passages. Middle row shows only Fos label. Fos-positive neurons have bright green within their nuclei. Bottom row shows an overlay of both WGA and Fos label. Scale bar in bottom left panel = 200 μm for left and middle columns; scale bar in bottom right panel = 100 μm for right column.

After injection of WGA into the left nasal passages of rats with intact AENs (N = 7), WGA label was located primarily in the ventral portion of the left MDH, between the pyramidal decussation caudally and obex rostrally (Figure 3). This roughly corresponds to a 1,500 µm rostral-caudal extent of the trigeminal nucleus caudalis (Table 1). Caudally, WGA label was only found in the ventral tip of the superficial MDH (laminae I and II) along the spinal trigeminal tract. Nearer to calamus scriptorius (the caudal end of the area postrema), the WGA label within the MDH shifted to a dorsolateral-ventromedial orientation with the appearance of spinal trigeminal nucleus interpolaris (Sp5I). As Sp5I became more prominent ventrolaterally, the WGA label was found more medially. More rostrally, nearer to obex, the WGA label in the MDH assumed a more dorsal-ventral orientation and began to break up. No label was found in deeper MDH locations (laminae III, IV, or V). WGA label was also found in the ipsilateral NTS, extending from calamus scriptorius caudally to rostral to obex rostrallly (Figure 4). At, and rostral to obex, WGA label was found along the dorsolateral edge of Sp5I adjacent to the ipsilateral trigeminal tract (Figure 4).

After injection of WGA into the left nasal passages of rats in which the AENs had been cut bilaterally (N = 3), WGA label was located in a more restricted range (approximately a 600 µm rostral-caudal extent; Table 1) primarily just caudal to obex. At more rostral locations the WGA label in the MDH was in a dorsal-ventral orientation adjacent to Sp5I. WGA label was also found in the ipsilateral NTS, and along the dorsolateral edge of Sp5I adjacent to the ipsilateral trigeminal tract.

**FIGURE 5:**
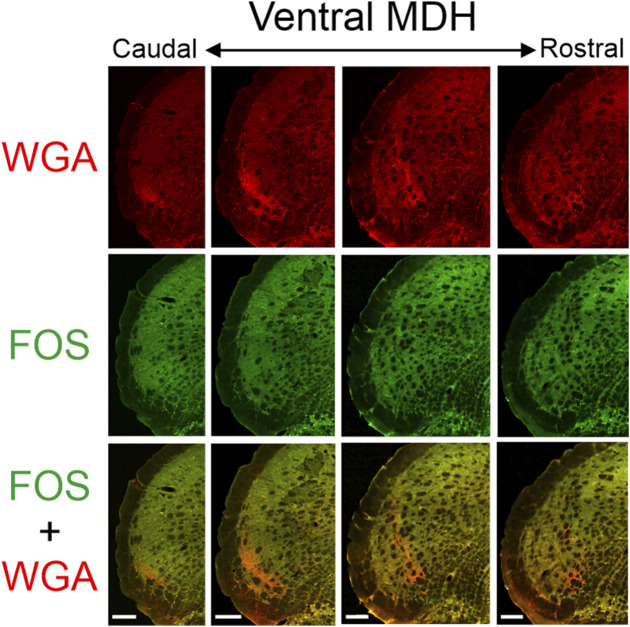
Low magnification confocal images of the left MDH from repetitively diving rats that had WGA injected into their left AENs. Images are maximum intensity projections and are arranged from caudal (left) to rostral (right). Top row shows only WGA label. The bright red shows the central terminations of the anterior ethmoidal nerve that innervates the nasal passages. Note how the label changes orientation when moving from caudal to rostral. Middle row shows only Fos label. Although difficult to see at this level of magnification, Fos-positive neurons have bright green within their nuclei. Bottom row shows an overlay of both WGA and Fos label. Note: no WGA label was found in either the NTS or dorsolateral edge of Sp5I adjacent to the ipsilateral trigeminal tract (not shown). Scale bar in bottom panels = 200 μm for that column. Images correspond to panel Gi in Figure 1.

**FIGURE 6:**
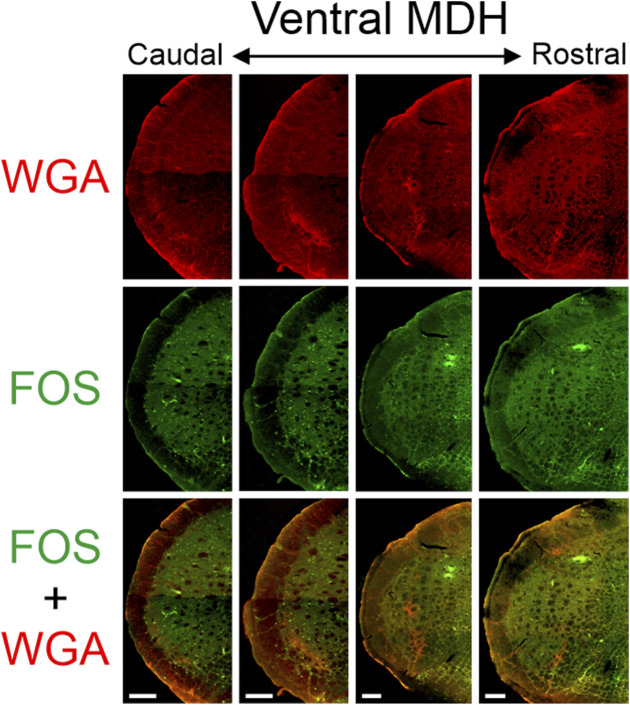
Low magnification confocal images of the left MDH from repetitively diving rats that had WGA injected into their left NPNs. Images are maximum intensity projections and are arranged from caudal (left) to rostral (right). Top row shows only WGA label. The bright red shows the central terminations of the nasopalatine nerve that innervates the nasal passages. Note how the label changes orientation when moving from caudal to rostral. Middle row shows only Fos label. Although difficult to see at this level of magnification, Fos-positive neurons have bright green within their nuclei. Bottom row shows an overlay of both WGA and Fos label. Note: no WGA label was found in the NTS but was found along the dorsolateral edge of Sp5I adjacent to the ipsilateral trigeminal tract (not shown). Scale bar bottom panels = 200 μm for that column. Images correspond to panel Gi in Figure 1.

After injection of WGA into the left AEN (N = 13; Figure 5) or left NPN (N = 7; Figure 6) the central WGA labeling pattern within the MDH was very similar to that seen after WGA injection into the nasal passages. WGA label was located primarily in the ventral portion of the left superficial MDH (laminae I and II), between the pyramidal decussation and obex. The label in the ventral tip of the MDH shifted to a more dorsal-ventral orientation, and moved medially, as Sp5I appeared. No label was found in deeper MDH locations (laminae III, IV, or V). In contrast to WGA injection into the nasal passages, after injection of WGA injection directly into individual nerves (AEN or NPN) WGA label was not found in the NTS. After injection of WGA injection directly into the NPN, WGA label was also found along the dorsolateral edge of Sp5I adjacent to the ipsilateral trigeminal tract near obex, and 2) caudally (in 2 of 7 rats) between the pyramidal decussation and calamus scriptorius. WGA label was never found dorsolaterally along the spinal trigeminal tract (Sp5), either rostrally or caudally, after injection of WGA into the AEN.

### 3.3 FOS-positive neurons

After rats engaged in repetitive diving, Fos-positive neurons within the MDH were primarily located between the pyramidal decussation and obex (Figures 3, 5, 6). Most caudally, Fos-positive neurons were found within the ventral tip of the MDH superficial laminae. Moving rostrally, the Fos-positive neurons were still found with the superficial MDH even as the MDH shifted medially with the appearance of Sp5I, and tended to be the most numerous between calamus scriptorius and obex. Fos-positive neurons were more prevalent in the MDH of diving animals compared to the MDH of non-diving control animals. It was noted that approximately 90% of all Fos-positive neurons within the MDH were located within the outlined area of WGA termination. However, the number of Fos-positive neurons within the WGA ROI per hemisection depended upon where the WGA had been injected, whether the AEN was intact or not, and whether the rat repetitively dived underwater or was a non-diving control (Table 1). Fos-positive neurons were located bilaterally in the MDH but were only counted in the left MDH hemisection (the location of the central terminations of the WGA injections).

**FIGURE 7:**
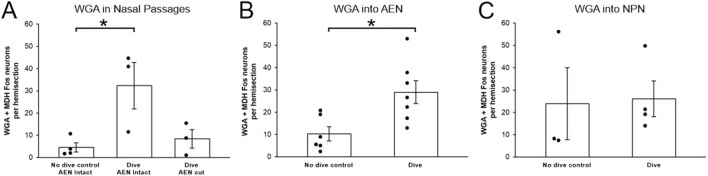
Fos-positive neurons within the WGA region of interest per hemisection before and after repetitive diving. Bar graphs show mean ± SE, while closed circles show results from individual animals. **(A)** After WGA was injected into the nasal passages of rats with intact AENs, there were significantly more Fos-positive neurons in rats that dived repetitively compared to no dive control rats. After WGA was injected into the nasal passages of diving rats in which the AENs had been cut bilaterally, there were fewer Fos-positive neurons compared to intact diving rats, although the difference was not significant. **(B)** After WGA was injected into the left AEN, there were significantly more Fos-positive neurons in rats that dived repetitively compared to no dive control rats. **(C)** After WGA was injected into the left NPN, there was not a significant difference in the number of Fos-positive neurons in diving rats compared to no dive control rats.

After injection of WGA into the left nasal passages, 4.6 ± 2.1, 32.3 ± 10.6, and 8.4 ± 4.2 Fos-positive neurons per hemisection (Table 1; Figure 7A) were found within the WGA ROI in non-diving control rats (N = 4), diving rats that had intact AENs (N = 3), and diving rats that previously had their AENs sectioned bilaterally (N = 3), respectively. A one-way ANOVA showed there were differences between these three groups (p = 0.027). A Holm-Sidak all pairwise multiple comparison procedures *post hoc* test showed significant differences between Dive AEN Intact and No dive control AEN Intact (p = 0.035), but not between Dive AEN Intact and Dive AEN cut (p = 0.059), nor between No dive control AEN Intact and Dive AEN cut (p = 0.657).

After injection of WGA into the left AEN, 10.3 ± 3.2 and 29.0 ± 5.2 Fos-positive neurons per hemisection (Table 1; Figure 7B) were found within the WGA ROI in non-diving control rats (N = 6) and diving rats (N = 7), respectively. A Student’s t-test showed these two groups were significantly different (p = 0.013).

After injection of WGA into the left NPN, 23.6 ± 16.3 and 26.1 ± 8.1 Fos-positive neurons per hemisection (Table 1; Figure 7C) were found within the WGA ROI in non-diving control rats (N = 3) and diving rats (N = 4), respectively. A Student’s t-test showed these two groups were not significantly different (p = 0.902).

After WGA injection into the nasal passages, AEN, or NPN, there were no differences in the number of Fos-positive neurons per hemisection within the WGA ROI between the three groups of non-diving control rats (one-way ANOVA, p = 0.225), or between the three groups of diving rats (one-way ANOVA, p = 0.865).

## 4 Discussion

In mammals, underwater submersion initiates the cardiorespiratory reflex known as the diving response. Part of the diving response central neuronal circuitry involves activation of neurons within the spinal trigeminal nucleus, specifically the MDH. The activation of neurons within the trigeminal nucleus would constitute the afferent limb of the circuit that receives and processes the initial sensory signal from the nose and nasal passages. The present study presents novel evidence to show that the location of MDH neurons activated by repetitive diving superimposes the same anatomical location that receives central terminations from the nasal passages. Thus, it is very likely that it is the afferent signals from the nasal passages that induce secondary neurons with the MDH to become activated during diving. These data also support previous suggestions that the anterior ethmoidal nerve, and possibly nasopalatine nerve, is involved in this process (McCulloch et al., 2018).

### 4.1 Diving behavior in rats

For air-breathing vertebrates, the most important aspect of the diving response is the cessation of respiration. Without an interruption of respiratory rhythmogenesis during submersion, water would be drawn into the lungs with each subsequent inspiratory effort. Some species exhale before or upon diving and maintain apnea during underwater submergence with low lung volumes; other species, including humans, submerge underwater after inspiration, thus maintaining apnea with large lung volumes (Snyder, 1983; Ponganis, 2011; Davis, 2014). Currently it is unknown whether conscious rats dive upon expiration or inspiration. However, electrical stimulation of the AEN in the rat working heart brainstem preparation (WHBP) causes central inspiratory neurons to cease firing and hyperpolarize, and central post-inspiratory neurons to depolarize and discharge continuously (Dutschmann and Paton, 2002). Thus voluntary submersion in rats presumably includes a post-inspiratory breath-hold.

#### 4.1.1 Air bubbles

This study is the first to report that rats expel air bubbles from their nose while diving. Contraction of the expiratory muscles, i.e., the abdominal muscles, moves air from the lungs through the upper airways to the nose. During submersion this would cause air bubbles to be expelled from the external nares. This suggests rats dive with respiratory expiration rather than respiratory inspiration. However, rats did not expel bubbles immediately upon diving when leaving the starting area. Instead, the majority of observed bubbles were produced when the rats were in the middle half of the underwater diving pathway. Consequently, this suggests rats exhibit expiratory efforts during diving, rather than diving upon expiration. Antarctic fur seals also exhale air bubbles during diving, but this occurs during ascent, which probably guards against shallow-water blackout (Hooker et al., 2005).

All rats formed air bubbles while diving, but not all rats formed air bubbles during every dive. This suggests air exhalation is not an obligatory occurrence during submergence. Mechanical contraction of abdominal muscles during underwater swimming movements may have caused air to emerge from the nose. Bubbles were most often observed while rats were rounding corners between dive channels. This physical maneuver presumably would cause further activation and contraction of abdominal muscles and may also have pushed abdominal contents into the diaphragm thus reducing thoracic volume. Either possibility would result in exhalation of air and the formation of bubbles. Thus, production of air bubbles while diving may be related to additional contraction of expiratory muscles during locomotory activity rather than the cessation of breathing and/or the production of a post-inspiratory breath-hold.

#### 4.1.2 Bloody noses

As noted previously (Chotiyanonta et al., 2013), rats occasionally had slightly bloody noses after some, but not all, diving trials. This was generally seen during early training sessions. Although speculative, the bloody noses, when present, were probably produced by water entering the nasal passages during diving, causing capillaries in the nasal passages to burst. Also, rats sniffed the water during head submersion while resting on the finishing area platform between diving trials. These sniffing movements would actively draw water into the nasal passages. Therefore rats apparently allow water to enter their internal nasal passages during underwater submersion. This is surprising (and somewhat counterintuitive) because drawing, or even allowing, water into the nasal passages during underwater submergence would be inherently dangerous, possibly leading to the lungs filling with water and subsequent drowning. However it is noteworthy that electrical stimulation of the AEN causes tonic activity of the recurrent laryngeal nerve in the rat WHBP, and thus it may be possible that diving animals routinely allow water to enter the nasal passages but prevent water entry into the lungs by producing glottal closure (Dutschmann and Paton, 2002). Additionally, exhalation of air bubbles during diving may have been an attempt to remove water from the nasal passages during underwater submergence.

#### 4.1.3 Role of the AEN

The nerve most often thought to carry the afferent signal from the nose and nasal passages to produce reflex breathing cessation is the AEN (McCulloch, 2012; Panneton, 2013). However, after AENs are cut bilaterally, diving rats neither drown nor draw water into their lungs. These rats still dive underwater, and exhibit dives behaviorally no different than dives from rats with intact AENs (present research; (Chotiyanonta et al., 2013; McCulloch et al., 2016)). Additionally, the AEN does not serve to prevent water entry into the nasal passages, as the frequency of bloody noses did not increase after bilateral AEN sectioning. Furthermore, air bubble production occurs with a similar frequency in rats diving both with and without intact AENs. This suggests that other nerves innervating the nose and nasal passages, besides the AEN, are also involved in the initiation of the respiratory and cardiovascular features of the diving response.

### 4.2 Central termination of nasal sensory afferents

After injection of WGA into the left nasal passages, AEN, or NPN, we found central WGA label in the same MDH locations as has been previously reported for rats (Anton and Peppel, 1991; Panneton et al., 2006; Hollandsworth et al., 2009; McCulloch et al., 2018), muskrats (Panneton, 1991), and cats (Lucier and Egizii, 1986). At the level of the pyramidal decussation the WGA was located primarily in the ventral tip of the left MDH, but by the level of the obex the label moved more medially and had a more dorsal-ventral orientation with the appearance of Sp5I.

After injection of WGA into the left nasal passages, we also found WGA label within the ipsilateral NTS, as has been described previously (Anton and Peppel, 1991; McCulloch et al., 2018). This WGA label was still found in the NTS in rats with bilaterally sectioned AENs, but was not found in the NTS after WGA was directly injected into the AEN (present study; (Panneton et al., 2006; Hollandsworth et al., 2009; McCulloch et al., 2018)). Thus, we agree with McCulloch et al. (2018) who concluded that NTS labeling from the nasal passages could not be due to the AEN and instead must be due to other nerves that innervate the internal nasal passages. The present research eliminates the NPN as a possible candidate for this NTS labeling, as WGA label was not present in the NTS after WGA was directly injected into the NPN. Alternatively, WGA injectate may have leaked proximally from the nasal passages into the nasopharynx which possibly may account for the observed NTS labeling (McCulloch et al., 2018).

After injection of WGA into the left nasal passages in rats both with and without intact AENs, we found WGA label along the left dorsolateral edge of Sp5I adjacent to the trigeminal track at and just rostral to obex, which has also been previously described (McCulloch et al., 2018). The NPN is likely the source of this label, as label was also found in this location after WGA was directly injected into the NPN but not after WGA was directly injected into the AEN (present study; (McCulloch et al., 2018)).

### 4.3 Activation of MDH neurons and the production of Fos

The *c-fos* gene and the Fos protein are markers of cellular activity and are easily expressed after a wide range of stimuli (Morgan and Curran, 1991; Coggeshall, 2005; Lara Aparicio et al., 2022). The Fos protein has a molecular weight of 56–62 kDa and is found in the nuclei of activated neurons (Lara Aparicio et al., 2022). While there are limitations to the technique, Fos has been studied in several experimental animal models since the 1990 s, and its use as a marker of cellular activation for many physiological processes, such as sensory information processing, is useful and reliable (Kovacs, 2008; Lara Aparicio et al., 2022). However, since Fos can be produced in numerous signaling pathways, its expression does not provide specific information about a given pathway, including inclusion of inhibitory processes (Dragunow and Faull, 1989; Coggeshall, 2005; Kovacs, 2008; Lara Aparicio et al., 2022). Immunohistochemistry is generally used to detect the presence of the Fos protein, and it is widely used to enable *in vivo* identification of activated cells (Morgan and Curran, 1991; Coggeshall, 2005; Kovacs, 2008; Lara Aparicio et al., 2022). Several studies have previously used the Fos technique to identify brainstem regions that become activated during diving (McCulloch, 2005; Panneton et al., 2010a; Panneton et al., 2012; McCulloch et al., 2016). It is assumed in the present study that when the rats engaged in voluntary diving the cardiovascular consequences of the diving response were activated. This seems likely, as previous studies using implantable blood pressure transmitters indicate that in all rats all underwater submersions initiate these cardiovascular responses (Panneton et al., 2010b; Chotiyanonta et al., 2013).

In the present study with repetitively diving rats, we found Fos-positive neurons located in the ventral tip of the caudal MDH between the pyramidal decussation caudally and the obex rostrally. As Sp5I appeared laterally, the location of the Fos-positive neurons shifted more medially. There were significantly more Fos-positive neurons in diving rats compared with non-diving control rats. The location of Fos-positive neurons within the MDH after repetitive diving in the present results is similar to that found previously (McCulloch, 2005; Panneton et al., 2010a; Panneton et al., 2012; McCulloch et al., 2016), and supports these previous studies. Additionally, the increases in Fos labelling within these MDH regions are seen during diving but not swimming (McCulloch, 2005; Panneton et al., 2010a; Panneton et al., 2012).

### 4.4 The afferent limb of the diving response circuit

A question posed in the present study was by what route does stimulation of the nasal passages during repetitive diving in rats cause MDH neurons to become active and produce Fos? McCulloch et al. (2018) previously found that afferent fibers from the internal nasal passages, but not external nasal region, project to the MDH in an anatomically appropriate manner to cause activation of secondary neurons within the ventral MDH during diving. In the present study there are striking similarities between the location of the central projections of WGA injected into the nasal passages (or nerves that innervate the nasal passages) and the location of Fos-positive neurons after repetitive diving. This close anatomical similarity of activated MDH neurons during diving and central terminations of nasal afferents has been noted previously (McCulloch, 2005; Panneton et al., 2010a; McCulloch, 2012; Panneton, 2013; McCulloch et al., 2016; McCulloch et al., 2018), but until now have never been specifically investigated.

**FIGURE 8:**
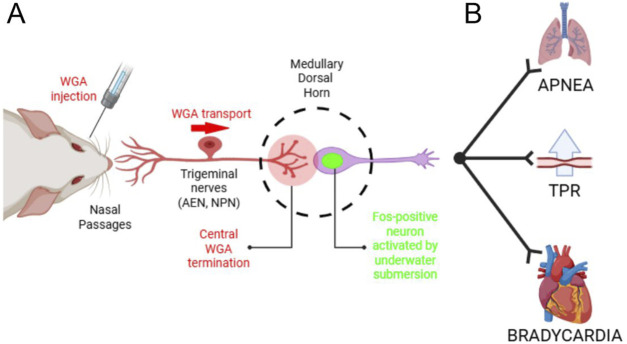
Summary of the investigated anatomical pathway. **(A)** After WGA was injected into the nasal passages, AEN or NPN, WGA transported centrally into the ventral portion of the MDH. In these same WGA injected rats, repetitive diving caused MDH neurons to become activated and express the Fos protein. In the present experiments we counted the number of Fos-positive MDH neurons located within the WGA termination field in both diving and non-diving control animals. Novel evidence shows that the location of MDH neurons activated by repetitive diving superimposes the same anatomical location that receives central terminations from the nasal passages. **(B)** Schematic pathway within the brainstem that accounts for the efferent aspects of the diving response, namely, apnea, a selective increase in peripheral vascular resistance, and bradycardia. For discussions on the brainstem circuitry that mediate the individual components of the diving response, see Panneton et al. (Panneton et al., 2000) and Panneton (Panneton, 2013). Figure created in BioRender.com.

The present results strongly suggest afferent fibers from the internal nasal passages project to the MDH and provide the appropriate sensory stimulation to induce neurons located there to produce Fos during repetitive diving (Figure 8). When comparing diving rats to non-diving control rats, there was a significant increase in the number of Fos positive neurons within the MDH that receive sensory information from the internal nasal passages as shown by WGA terminal projections. Thus these secondary MDH neurons likely were activated after receiving afferent signals from the nasal passages during diving. In diving rats that had their AENs sectioned bilaterally along with injections of WGA into the nasal passages, there were far fewer Fos positive neurons within the MDH. We also found there was a significant increase in the number of Fos positive neurons within the area of the MDH receiving terminal projections from the AEN of diving rats compared to non-diving control rats. Collectively, these data strongly suggest that the AEN is important for carrying the appropriate sensory information to the MDH to induce the secondary neurons within the MDH to become activated and produce Fos during repetitive diving.

Other trigeminal nerves, besides the AEN, that may also be involved in initiating the diving response are the infraorbital nerve (Panneton, 2013) and nasopalatine nerve (McCulloch et al., 2018). McCulloch et al. (2018) found the NPN, but not the peripheral branches of the infraorbital nerve, has the appropriate central terminations that could possibly also provide sensory information to the MDH to induce these neurons to become activated and produce Fos during repetitive diving. To investigate this possibility further, we injected WGA directly into the NPN of rats trained to dive. In these rats we found the number of Fos positive neurons within the area of the MDH receiving terminal projections from the NPN was not significantly different from the MDH of diving rats that had WGA injected into either the nasal passages or directly into the AEN. However, we found in non-diving rats receiving injections of WGA into the NPN that the number of Fos-positive neurons was much greater than non-diving control rats having had WGA injections into either the nasal passages or directly into the AEN. Thus the reason the number of Fos-positive neurons in the MDH of rats receiving injections of WGA into the NPN was not significantly greater in diving rats compared to non-diving rats was primarily because the non-diving control rats had an unexpectedly high number of Fos-positive neurons. We are unsure of why these non-diving control rats had such a high number of Fos-positive neurons after WGA injections into the NPN. However, it is possible that the more invasive surgery necessary to access the NPN deep within the orbit, and/or possible irritation of the overlying main bundle of the infraorbital nerve during the surgery, may have caused residual activation of this nerve that secondarily activated neurons within the MDH and induced them to produce Fos even when not engaging in repetitive diving. In any event, based on the present data we cannot conclude that the NPN provides sensory information to the MDH to induce MDH neurons to produce Fos during repetitive diving.

A limitation of the present study, however, is that anatomical appropriateness does not prove causality. For instance, terminal WGA projections, however suitable, do not provide evidence that these sensory terminations were actually responsible for inducing MDH neurons to become activated and produce Fos during diving. Consequently, we are only able to report the number of Fos-positive neurons within the WGA ROI as opposed to double-labeled neurons. While it does seem very likely that these nasal terminations are responsible for activating the MDH neurons, electrophysiological evidence would be necessary to prove it. A second limitation of this study is that we only counted Fos-positive neurons within the WGA ROI, and not in nearby regions that can also show Fos-positive neurons after diving (i.e., spinal dorsal horn, paratrigeminal nucleus, and deep MDH laminae–see McCulloch (2005)). However, the vast majority of Fos-positive MDH neurons were indeed found within the WGA termination field, which likely represents the critical site responsible for the initial sensory processing of information received from the nasal passages. However, even with these limitations, we are confident to conclude that sensory information from the nasal passages, projecting specifically along the AEN (and perhaps even the NPN) are responsible for activating MDH neurons and inducing them to produce Fos during repetitive diving.

## 5 Conclusion

In summary, we trained rats to repetitively dive underwater to investigate the route by which afferent information causes MDH neurons to become active and produce Fos during underwater submergence. In repetitively diving rats that had previously received WGA injections into the left nasal passages, AEN, or NPN, both Fos-positive MDH neurons and central WGA label were found in the ventral tip of the left MDH between the pyramidal decussation caudally and the obex rostrally. Although anatomical appropriateness does not prove causality, we strongly suggest that the overlapping labeling patterns of Fos-positive neurons and WGA provide evidence that sensory information from the nasal passages, projecting specifically along the AEN (and possibly the NPN), is responsible for activating MDH neurons and inducing them to produce Fos during repetitive diving.

In addition, we found that rats often expel air bubbles from their nose during repetitive diving. However, this bubble formation may have had more to do with contraction of expiratory muscles rather than part of a process to inhibit respiratory activity during the submergence. Furthermore, the observation of diving rats occasionally having bloody noses suggests that rats apparently allow water to enter their internal nasal passages during underwater submersion. Both bubble formation and bloody noses appear with equal frequency before and after bilateral sectioning of the AENs. Consequently, other nerves, perhaps the NPN, may also provide sensory information during diving.

## Data Availability

The raw data supporting the conclusions of this article will be made available by the authors, without undue reservation.

## Abbreviations

AEN, anterior ethmoidal nerve; BSA, bovine serum albumin; IHC, immunohistochemistry; MDH, Medullary Dorsal Horn; NPN, nasopalatine nerve; NTS, nucleus tractus solitarius; PBS, phosphate buffered saline; ROI, region of interest; WGA, wheat germ agglutinin.
